# Impact of meniscus injury and chondromalacia on the patient‑reported quality of life, social support, and mental health following knee arthroscopy

**DOI:** 10.20452/wiitm.2025.17923

**Published:** 2025-01-03

**Authors:** Bartosz Turoń, Damian Małkowski, Maria Zabrzyńska, Krzysztof Buczkowski, Piotr Błaszak, Jakub Ohla, Michał Wilk, Janet Olagbaju, Agnieszka Witowska, Jan Zabrzyński

**Affiliations:** Department of Orthopaedics and Traumatology, Regional Hospital, Grudziądz, Poland; Department of Family Medicine, Collegium Medicum, Collegium Medicum, Nicolaus Copernicus University in Torun, Toruń, Poland; Department of Orthopaedics and Traumatology, Faculty of Medicine, Collegium Medicum in Bydgoszcz, Nicolaus Copernicus University in Torun, Bydgoszcz, Poland; Department of Metabolism, Endocrinology, and Internal Medicine, Poznan University of Medical Sciences, Poznań, Poland

**Keywords:** arthroscopy, chondromalacia, meniscus injury, quality of life

## Abstract

**INTRODUCTION:**

Meniscal tears and chondromalacia patellae (CP) are among the most common knee pathologies treated with arthroscopic partial meniscectomy. Chronic knee pain impairs mobility, functionality, and the overall quality of life (QOL).

**AIM:**

The study evaluates the relationship between meniscus injury and early-stage CP found intraoperatively, and pre- and postoperative patient-reported QOL, perceived social support, and mental health. MATERIALS AND METHODS Patients who underwent knee arthroscopy for meniscal and cartilage abnormality, without reconstructive or restorative procedures, between 2019 and 2021, were prospectively enrolled in this study.

**METHODS:**

Patients who underwent knee arthroscopy for meniscal and cartilage abnormality, without reconstructive or restorative procedures, between 2019 and 2021, were prospectively enrolled in this study.

**RESULTS:**

We observed a significant rise in the pre- and postoperative scores on the World Health Organization QOL Brief Version (WHOQOL-BREF) scale for various meniscal tears and different types of CP (grade 0–IV) in almost all domains. Education level did not significantly affect the WHOQOL-BREF assessment, and we found no statistical correlation between preoperative WHOQOL-BREF score in all domains and waiting time for surgery. However, there were significant differences between white- and blue-collar employees in the domains 1 and 2, that is, physical and psychological health.

**CONCLUSIONS:**

The study shows that the measured QOL improves in patients operated at the early stages of CP. Moreover, when both menisci are involved in the pathological process, the clinical outcomes are inferior. The education level and waiting time for surgery had no impact on QOL, contrary to the type of work, as white-collar workers had better outcomes in physical and psychological health domains.

## INTRODUCTION

Meniscal tears and chondromalacia patellae (CP) are among the most frequently encountered knee pathologies in orthopedic practice, with arthroscopic partial meniscectomy being the most commonly performed orthopedic procedure globally.^1^^,^^2^^,^^3^ Significance of these conditions is emphasized by a critical role of the menisci in the knee functioning. The menisci play a vital role in load distribution, joint stability, and shock absorption.^4^^,^^5^^,^^6^ Their damage can result in pain, tenderness, swelling, and mechanical symptoms, such as knee catching or locking.^7^^,^^8^ A study by Englund et al^9^ found that 63% of individuals experiencing knee pain, aching, or stiffness had a meniscal tear, indicating that such tears are of frequent occurrence. CP, on the other hand, is a condition characterized by deterioration, softening, and ulceration of the articular surface of the patella.^1^^,^^10^ Both conditions can cause pain, swelling, and limit the range of motion, consequently undermining the ability to perform daily activities and overall functional capacity.^11^^,^^12^^,^^13^

**TABLE 1:** Summary of demographic and clinical characteristics of patients

Parameter		Value
Patients		274
Age, y		43.54 (38-55)
Sex	Men	193
	Women	81
Education	Primary	11
	Secondary	100
	Tertiary	69
	Higher	94
Type of job	Mental	106
	Physical	168
Waiting time for surgery, d		13.43 (0-140)
Previous surgery		0

Data are provided as number or median and interquartile range.

**TABLE 2:** Summarized statistical analysis based on the preoperative and postoperative World Health Organization Quality of Life scale in patients with various types of meniscal tears

Type of meniscal tear	Domain	Preoperative WHOQOL score, mean (SD)	Postoperative WHOQOL score, mean (SD)	P value
Both menisci tear	Physical health	63 (10.81)	76.75 (6.5)	0.006
	Psychological health	80.82 (7.29)	88.32 (7.93)	0.03
	Social relationships	80.83 (5.56)	84.87 (9.96)	0.49
	Environment	77.25 (4.52)	82.25 (5.15)	0.07
Lateral meniscus tear	Physical health	58 (10.19)	70.63 (7.64)	<0.001
	Psychological health	74.81 (9.44)	84.63 (7.64)	0.001
	Social relationships	76.04 (7.9)	79.51 (6.75)	0.13
	Environment	74.92 (6.66)	78.25 (6.64)	<0.001
Medial meniscus tear	Physical health	57.69 (11.21)	71.87 (11.15)	<0.001
	Psychological health	74.23 (10.19)	83.10 (10.35)	<0.001
	Social relationships	55.77 (11.16)	79.85 (10.39)	<0.001
	Environment	74.38 (10.26)	78.42 (9.82)	<0.001

Abbreviations: WHOQOL, World Health Organization quality of life scale

These diseases not only hinder mobility and functionality, but also significantly affect the overall quality of life (QOL). The correlation between chronic pain and mental health disorders is widely recognized, with patients who suffer from chronic pain being more likely to exhibit signs of depression, anxiety, and social withdrawal.^14^ Meniscal tears have been associated with reduced physical function, higher pain levels, and poorer QOL.^9^^,^^15^ Similarly, CP has been linked to reduced physical activity and limited mobility, as well as lower levels of emotional well‑being, social functioning, and overall health, as compared with the general healthy population.^16^ Studies have shown that increased social support is crucial for enhancing the overall QOL of individuals affected by physical limitations and chronic pain.^17^^,^^18^ Furthermore, those who have consistent and reliable social support networks have demonstrated an enhanced ability to effectively cope with challenges and stressors, thus diminishing their susceptibility to depression, anxiety, and social isolation.^19^^,^^20^^,^^21^ The impact of both CP and meniscus injuries on QOL should not be underestimated. It requires additional research to understand the correlation between these knee conditions, mental well‑being, and perceived social support.

By analyzing multiple dimensions such as age, educational background, and type of work performed by the patients, this research endeavors to gain valuable insights into the overall impact of knee pathologies on different demographics, thus allowing a more nuanced understanding of the difficulties experienced by patients, going beyond just physical symptoms. The results of this study could have significant implications for the holistic treatment of individuals with meniscus injuries and CP. By considering not only the physical aspects but also the psychosocial dimensions, health care providers can develop well‑rounded strategies that comprehensively address the needs of patients, ultimately improving patient outcomes and overall wellbeing.

## AIM

The aim of the study was to evaluate the influence of meniscus injury and early‑stage CP on pre‑ and postoperative patient‑reported QOL, perceived social support, and mental health.

## MATERIALS AND METHODS

This prospective study included patients who underwent knee arthroscopy for meniscal and cartilage abnormality, without reconstructive or restorative procedures, between 2019 and 2021. It was conducted by 2 senior orthopedic surgeons in the Department of Surgery at the Regional Specialist Hospital in Grudziądz, Poland.

The exclusion criteria were prior surgery on the ipsilateral or contralateral knee other than knee arthroscopy for partial meniscectomy and / or chondroplasty, an intraoperative diagnosis of an anterior cruciate ligament injury, or concomitant procedures including ligamentous repair or reconstruction, osteotomy, removal of hardware, irrigation, and debridement, microfracture, autologous chondrocyte implantation, or osteochondral autograft or allograft transfer. Moreover, the patients with axial knee deformity and radiological signs of osteoarthritis who had arthroscopic debridement for isolated torn medial meniscus were excluded from the study. 

Clinically, the included patients had torn medial or lateral meniscus, pain in the knee, with or without associated mechanical symptoms not relieved by conventional methods, supported by magnetic resonance imaging (MRI) positive result and weight‑bearing radiographs with Kellgren and Lawrence grade 0 or 1 at the baseline visit.

**TABLE 3:** Summarized statistical analysis based on the preoperative and postoperative World Health Organization Quality of Life scale in patients with various grades of chondromalacia

Chondromalacia grade	Domain	Preoperative WHOQOL score, mean (SD)	Postoperative WHOQOL score, mean (SD)	*P* value
0	Physical health	58 (8.7)	72.71 (10.8)	<0.001
	Psychological health	75.71 (10.49)	84.76 (6.87)	0.006
	Social relationships	72.85 (6.87)	80.57 (6.24)	0.02
	Environment	77.07 (9.1)	80.64 (6.57)	0.17
1	Physical health	58.83 (10.81)	72.73 (9.95)	<0.001
	Psychological health	74.73 (10.7)	80.59 (8.91)	<0.001
	Social relationships	78.03 (10.19)	81.65 (9.49)	0.02
	Environment	75.28 (9.23)	79.07 (8.17)	0.002
2	Physical health	57.35 (11.04)	70.94 (11.24)	<0.001
	Psychological health	57.35 (11.04)	79.82 (11.57)	<0.001
	Social relationships	74.61 (11.57)	79.24 (11.5)	0.02
	Environment	74.32 (10.35)	78.27 (9.96)	0.001
3	Physical health	58.25 (12.19)	71.64 (10.23)	<0.001
	Psychological health	75.22 (10.02)	80.39 (9.81)	0.006
	Social relationships	75.29 (9.16)	79.49 (9.11)	0.051
	Environment	73.9 (9.32)	78.31 (10.23)	0.009
4	Physical health	55.84 (11.14)	71.10 (13.78)	<0.001
	Psychological health	73.14 (9.89)	78.06 (9.24)	0.02
	Social relationships	69.82 (10.96)	76.15 (11.3)	0.09
	Environment	70.52 (10.83)	75.63 (11.92)	0.03

Abbreviations: see TABLE 2

On the day of surgery, the patients were asked preoperatively to complete a questionnaire comprising sociodemographic data (including demographic information, such as sex, age, education level, type of work [mental or physical], waiting time for surgery, and previous surgery), and were examined using QOL‑measuring tools.

General QOL was assessed by the validated Polish version of the World Health Organization QOL Brief Version scale (WHOQOL‑BREF), which measures QOL with 26 questions in 4 domains: 1) physical health (activities of daily living, dependence on medicinal substances and medical aids, energy and fatigue, mobility, pain and dis‑ comfort, sleep and rest, and work capacity);b2) psychological health (bodily image and appearance, negative feelings, positive feelings, self‑esteem, spirituality / religion / personal beliefs, thinking, learning, memory, and concentration); 3) social relationships (personal relationships, social support, and sexual activity); 4) environment (financial resources, freedom, physical safety and security, health and social care: accessibility and quality, home environment, opportunities for acquiring new in‑ formation and skills, participation in and opportunities for recreation / leisure activities, physical environment, and transport).^22^As per the instructions in the WHOQOL‑BREF manual, domain scores were calculated and converted to a 0–100 scale. A higher score represented better QOL.

All preoperative evaluations and activities were undertaken by 2 senior orthopedic surgeons (BT, DM) experienced in knee arthroscopy and working in the same unit. Surgery was performed under general or intraspinal anesthesia with the patient in the supine position. A leg holder and tourniquet were placed around the thigh of the affected leg. Standard anterolateral and anteromedial knee portals were used. Diagnostic arthroscopy was performed to eval‑ uate abnormal findings. Medial meniscal tears were trimmed to a stable rim. During surgery, cartilage lesions were probed, measured, and then graded according to the International Cartilage Repair Society (ICRS) classification. The severity of chondral damage was determined by the ICRS grade and the number of compartments involved. The meniscus lesion data were cross‑referenced with the WHOQOL‑BREF score.

### Statistical analysis

All group comparisons and statistical analyses were conducted by 2 independent investigators using the Graph‑ Pad, Prism software (GraphPad 8.0.1, Dotmatics, London, United Kingdom). A P value below 0.05 was considered significant. The normality of variable distribution was assessed using the Shapiro–Wilk test. Descriptive statistics including frequency, percentage, mean, and SD were performed to characterize the study sample. As the outcome measures had a non‑normal distribution, the Wilcoxon tests were carried out to assess the difference in the WHOQOL out‑ comes measured pre‑ and postoperatively in various meniscal lesions and CP groups according to ICRS. The Kruskal–Wallis test was used to assess the difference in the WHOQOL outcomes measured before and after surgery for various education levels and age groups. The association between the waiting time for surgery and values for the WHOQOL domains were evaluated using the Spearman rank correlation coefficient.

### Ethics

This study was approved by a local institutional review board (523/2019). All participants provided their written informed consent.

## RESULTS

The study involved 274 patients. Their mean (SD) age at enrollment was 43.54 (11.06) years (range, 19–61 years). There were 81 wom‑ en and 193 men. The demographic data are presented in TABLE 1

**FIGURE 1:**
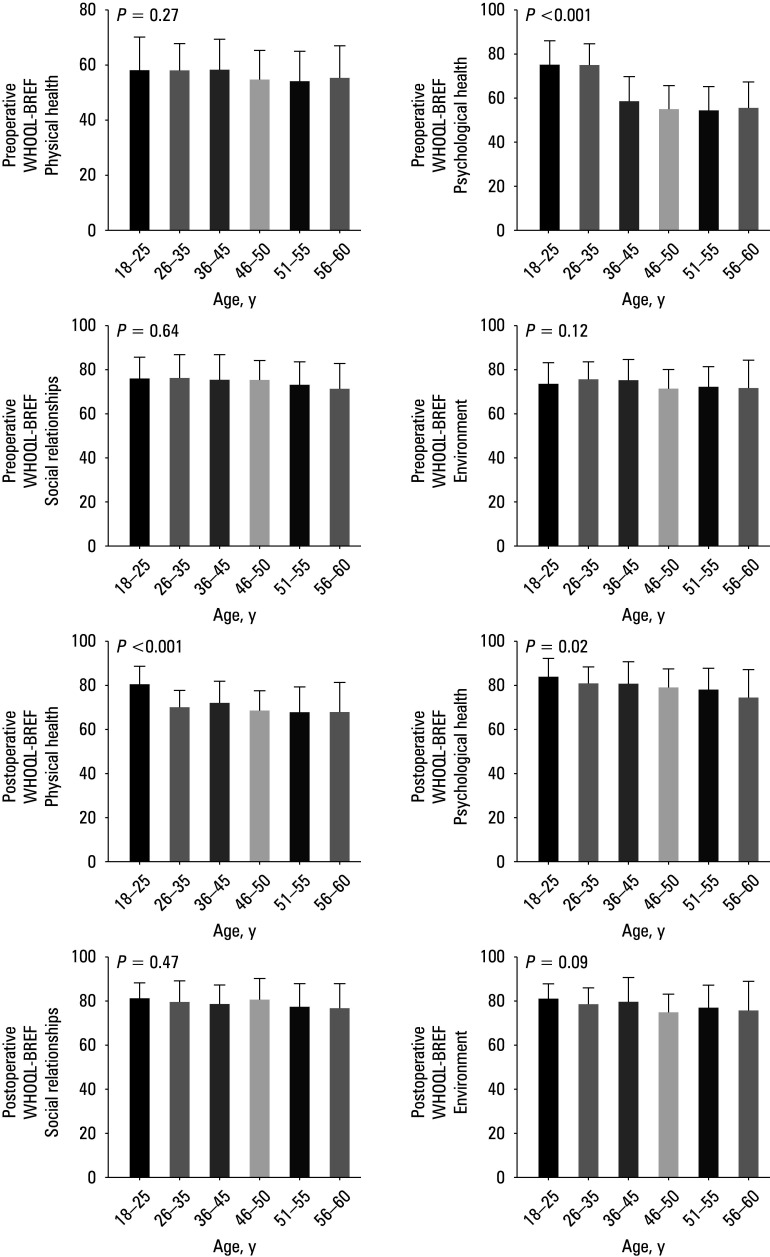
Associations between pre- and postoperative WHOQOL- -BREF scores and age of patients; A–D – preoperative WHOQOL -BREF results for age and the domain 1 (A), 2 (B), 3 (C), and 4 (D); E–H – postoperative WHOQOL -BREF results for age and the domain 1 (E), 2 (F), 3 (G), and 4 (H) Abbreviations: WHOQOLBREF, World Health Organization quality of life scale, brief version

**FIGURE 2:**
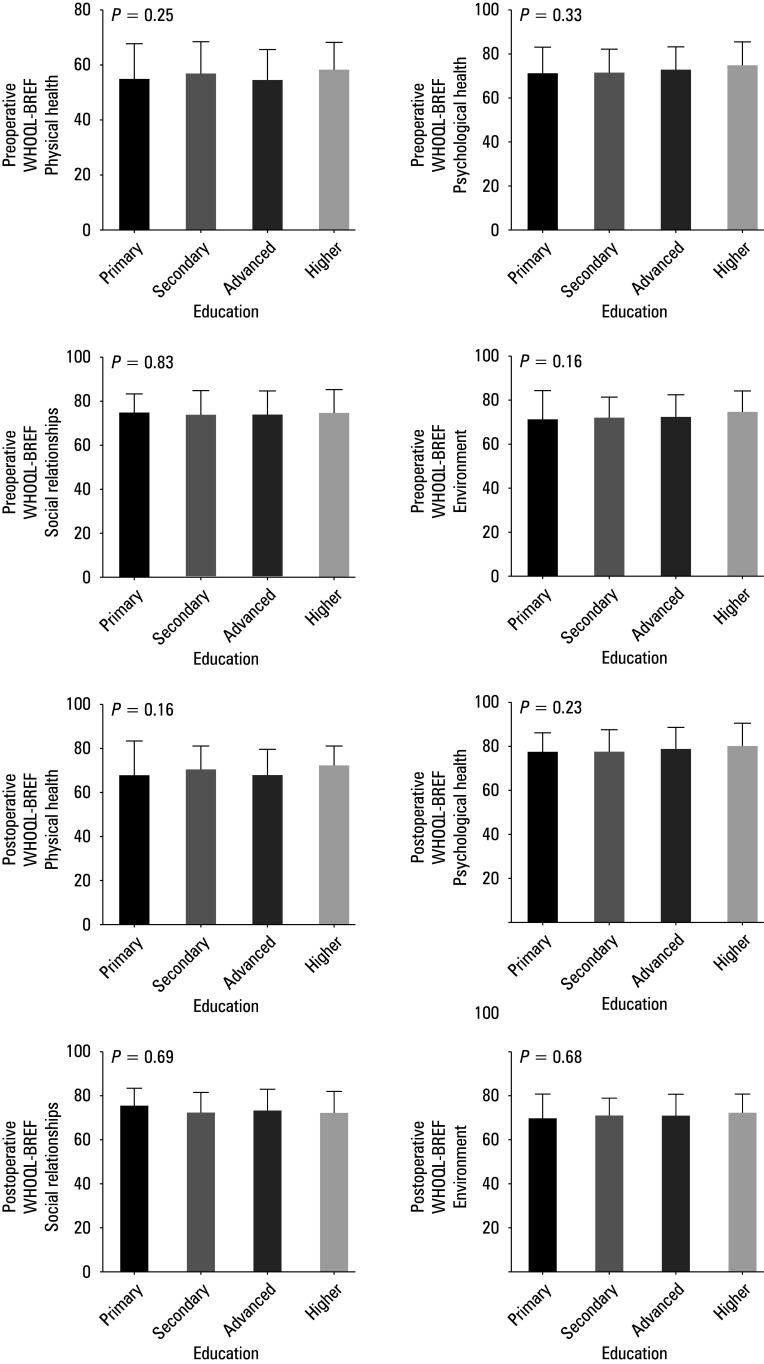
Summarized statistical analysis based on the preoperative and postoperative WHOQOL- -BREF scale and the level of education. A–D – preoperative WHOQOL -BREF results for the level of education and the domain 1 (A), 2 (B), 3 (C), and 4 (D); E–H – postoperative WHOQOL -BREF results for the level of education and the domain 1 (E), 2 (F), 3 (G), and 4 (H)

Abbreviations: see FIGURE 1

The patients were divided into 3 groups: 239 with damaged medial meniscus, 27 with damaged lateral meniscus, or 8 with both types of injury. Mean (SD) age of the patients with both menisci damaged was 46.12 (6.5) years (range, 38–55 years), with damaged lateral meniscus 41.63 (12.75) years (range, 19–60 years), and with damaged medial meniscus 43.68 (10.99) years (range, 19–61 years).

We found significant differences for all domains of the WHOQOL‑BREF questionnaire completed before and after surgery TABLE 2 and TABLE 3.

When comparing the pre‑ and postoperative WHOQOL‑BREF results in the group of patients with damaged both menisci, no signifi‑ cant differences were found in the domains 3 and 4 TABLE 2. In the group with damaged lateral meniscus, significant differences were identified in the domains 1, 2, and 4, and in the group with damaged medial meniscus, the differences were significant in all domains TABLE 2.

Furthermore, we observed significant differences in the pre‑ and postoperative WHOQO‑BREF scores in the domains 1, 2, and 3 but not domain 4 in the patients without CP TABLE 3. The pre and postsurgery differences in the WHOQOL‑BREF values were significant for all domains in the patients with first‑ and second‑grade CP TABLE 3, while for third‑ and fourth‑grade CP the differences were significant for the domains 1, 2, and 4 TABLE 3.

**FIGURE 3:**
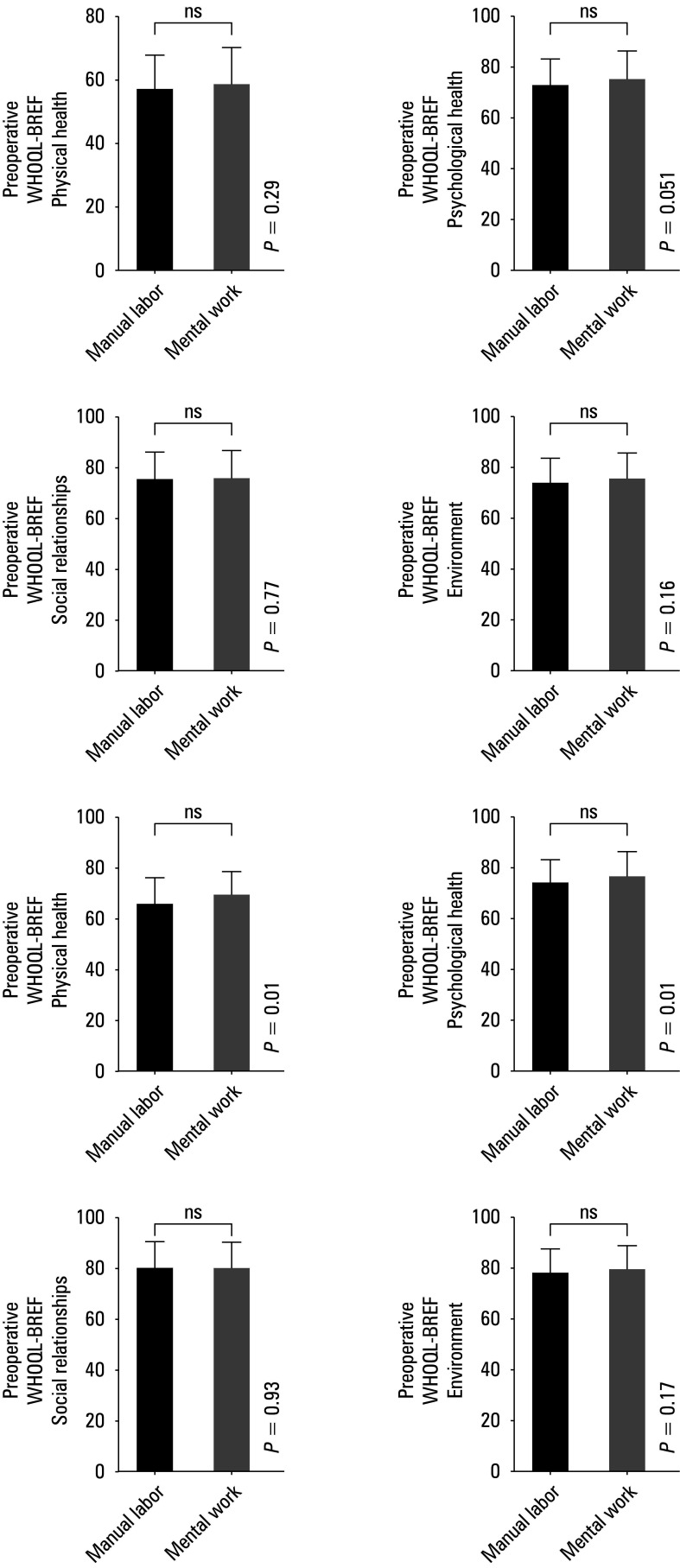
Summarized statistical analysis based on the preoperative and postoperative WHOQOL -BREF scale and the type of work A–D – preoperative WHOQOL- -BREF results for the type of work and the domains 1 (A), 2 (B), 3 (C), and 4 (D); E–H – postoperative WHOQOL -BREF results for the type of work and the domains 1 (E), 2 (F), 3 (G), and 4 (H)

Abbreviations: ns, nonsignificant; others, see FIGURE 1

Significant differences were found in the do‑ main 2 for the age of the patients FIGURE 1, but the level of education did not correlate with pre‑ and postoperative WHOQOL‑BREF results FIGURE 2.

There were no preoperative differences be‑ tween the mental and physical workers in the WHOQOL‑BREF domains or postoperative differences in the domains 3 and 4, but we observed significant differences between these groups in the domains of physical and physio‑ logical health FIGURE 3. No correlation was as‑ certained in either domain for the waiting time for surgery 

## DISCUSSION

The most important finding of this study was that endoscopic knee surgery may have a major positive impact on certain domains of QOL in the patients with knee meniscus inju‑ ry and early‑stage CP. However, results regard‑ ing the impact of CP and meniscus injury type on the postoperative outcomes differed greatly in the assessed domains. The patients without knee CP showed no significant differences in pre‑ and postsurgery scores in the domain 4 reflecting their environment. The patients with more ad‑ vanced stages of CP (III–IV) had similar pre‑ and postoperative values for the domain 3 reflecting their social relationships. Those with both me‑ nisci rupture demonstrated no improvement in the domains 3 and 4 after surgery. The education level had no significant impact on pre‑ and post‑ operative QOL, contrary to the type of work that showed significant differences for the domains 1 and 2, that is, physical and physiological health, respectively. Interestingly, waiting time for surgery did not correlate with QOL.

Knee pain is a factor negatively affecting QOL and leading to disability. The most common rea‑ sons for knee pain are osteoarthritis, patellofemoral pain, and meniscal tears.^23^^,^^24^ CP often leads to patellofemoral pain syndrome and disruption of patellar cartilage, which increases pressure on the articular surfaces and results in patellofem‑ oral cartilage loss leading to joint instability and biomechanical disturbances.^25^In young patients, knee pain due to patellofemoral joint pathologies is predominant and mostly indicates CP, and to a lesser extent ligament and meniscus injuries. It is worth noting that CP is not often observed on MRI, and physical examination is a preferred di‑ agnostic method. Not repairing the damaged cartilage can lead to the development of osteoarthritis.^26^ Cheung et al^27^ pointed out the importance of addressing psychosocial factors when formulating rehabilitation strategies for athletes with patellofemoral pain and their impact on physical aspects of QOL.

**FIGURE 4:**
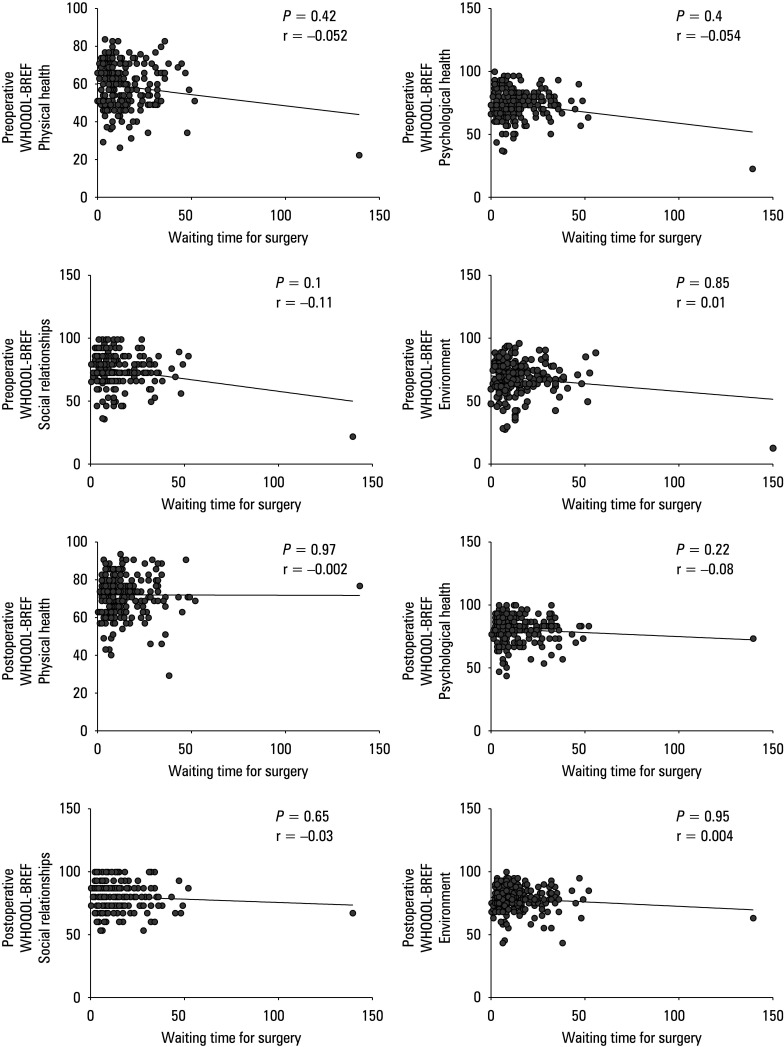
Summarized statistical analysis based on the preoperative and postoperative WHOQOL -BREF scale and waiting time for surgery. A–D – preoperative WHOQOL -BREF results for waiting time for surgery and the domain 1 (A), 2 (B), 3 (C), and 4 (D); E–H – postoperative WHOQOL -BREF results for waiting time for surgery and the domain 1 (E), 2 (F), 3 (G), and 4 (H)

Abbreviations: see FIGURE 1

The WHOQOL‑BREF scale is perceived as a proper tool to assess changes in the QOL following knee surgery. Wojcieszek et al^22^ noted that people without lower limb complaints obtained better results on the WHOQOL‑BREF scale than those with gonarthrosis. In our study, the QOL measured with the WHOQOL‑BREF scale improved significantly in every aspect of life in the patients with lower‑grade (I–II) CP. In‑ terestingly, no positive correlations were found for the domain 3 in the patients with third‑ and fourth‑grade CP. This means that for higher‑grade CP social relationships did not improve postoperatively, as opposed to physical and psychological health and environmental factors. Ackerman et al^28^ reported no significant differences in the WHOQOL‑BREF scale of social relationships after knee replacement, which may be due to a lack of change in the relationships in early postoperative period or to poor patient responsiveness in this domain. On the other hand, Mezey et al^29^ noted that surgical treatment of osteoarthritis significantly increased the perceived QOL, but only in the physical health and social relationship domain. In addition, Jaiswal et al^30^ demonstrated that knee osteoarthritis most strongly affected the physical and psychological domains of QOL, though social and environ‑ mental domains also improved considerably. Although the improvement in the QOL was small‑ er for third‑ and fourth‑grade CP, it was equally important as for the first and second‑grade due to developing osteoarthritis in advanced CP stag‑ es and total loss of self‑repair ability.^31^

Medial meniscal tears are one of the most common injuries of the knee, together with knee cartilage injuries most often concerning the me‑ dial femoral condyle and patellar articular surface.^32^Meniscal tears have become a more wide‑ spread injury in all age groups due to either trauma or osteoarthritis.^33^Both menisci undergo pathological changes, but they are more often in the medial meniscus.^34^ Bryceland et al^35^ observed that meniscal repair techniques are improving, and success rates are higher when surgery is performed early. Our study demonstrated improved QOL in all areas in the patients undergoing medial meniscus injury repair. The patients with lateral meniscus tear had no improvement in the social relationships. Moreover, the patients with tears of both menisi had poorer scores in the social relationship and environment domains. It is worth noting that the lateral meniscus bears around 70% of the lateral load, in contrast with the medial meniscus, which carries only 50% of the load.^34^^,^^36^ This can be the rea‑ son for difficulties in obtaining better outcomes of surgery, similarly to cases when both menisci are injured.

Patients aged 18–25 years have much better outcomes in physical health than older individuals. Fedje‑Johnston et al^37^ demonstrated that age is associated with a decrease in meniscal vessel count. Blood supply to the menisci is crucial for potential healing.^34^ Michel et al^38^ claimed that vascular anatomy is especially important, as it is thought to be one of the key factors in the heal‑ ing of meniscal tears, and reduced microvascular density can result in delayed or complicated wound healing.

Our study also investigated the impact of job profile on the QOL after meniscus surgery. Patients performing hard physical work are more prone to cartilage degeneration and loss, so they can benefit most from surgery.^29^ Mezey et al^29^ observed that patients with manual jobs reported significantly greater improvement in the physical and psychological health domains following surgical treatment of osteoarthritis than those in‑ volved in office work. Perry et al^39^ reported that heavy manual work carries an increased risk of incident knee osteoarthritis, particularly among men. Our study did not show a significant improvement in postoperative physical and psychological health domain in the blue‑collar workers, which was observed in the patients with office jobs, despite the fact that men predominated in the study group.

Several limitations were noted in this study. First, the patients were predominantly men with medial meniscus injuries. Second, first‑ and second‑grade CP prevailed. Third, the use of the WHOQOL‑BREF score represents a subjective measure, and understanding of the improvement in the QOL in various domains may vary among individuals. This could potentially introduce bias in our results concerning the WHOQOL‑BREF score. Finally, our statistical analyses were performed as unidimensional.

## CONCLUSIONS

The study shows that the mea‑ sured QOL improves in patients operated at early stages of CP. Moreover, when both menisci are involved in the pathological process, the clinical outcomes are worse. The level of education and waiting time for surgery had no impact on QOL, contrary to the type of job. Mental workers had better outcomes in physical and psychological health domains.
